# Goal Setting for Cognitive Rehabilitation in Mild to Moderate Parkinson's Disease Dementia and Dementia with Lewy Bodies

**DOI:** 10.1155/2016/8285041

**Published:** 2016-06-29

**Authors:** Tamlyn J. Watermeyer, John V. Hindle, Julie Roberts, Catherine L. Lawrence, Anthony Martyr, Huw Lloyd-Williams, Andrew Brand, Petra Gutting, Zoe Hoare, Rhiannon Tudor Edwards, Linda Clare

**Affiliations:** ^1^School of Psychology, Bangor University, Bangor LL57 2AS, UK; ^2^Department of Care for the Elderly, Betsi Cadwaladr University Health Board, Llandudno LL30 1LB, UK; ^3^The College of Health & Behavioural Sciences, Bangor University, Bangor LL57 2UW, UK; ^4^Division of Mental Health and Learning Disabilities, Betsi Cadwaladr University Health Board, Ysbyty Gwynedd, Bangor LL57 2PW, UK; ^5^Centre for Research in Ageing and Cognitive Health (REACH), School of Psychology, University of Exeter, Exeter EX4 4QG, UK; ^6^PenCLAHRC, Institute of Health Research, University of Exeter Medical School, Exeter EX1 2LU, UK; ^7^Centre for Health Economics and Medicines Evaluation (CHEME), Bangor University, Normal Site, Bangor LL57 2PZ, UK; ^8^The North Wales Organisation for Randomised Trials in Health (NWORTH), Bangor University, Gwynedd, Bangor LL57 2PZ, UK; ^9^Cefni Hospital, Llangefni, Anglesey LL77 7PP, UK

## Abstract

Alongside the physical symptoms associated with Parkinson's disease dementia and dementia with Lewy bodies, health services must also address the cognitive impairments that accompany these conditions. There is growing interest in the use of nonpharmacological approaches to managing the consequences of cognitive disorder. Cognitive rehabilitation is a goal-orientated behavioural intervention which aims to enhance functional independence through the use of strategies specific to the individual's needs and abilities. Fundamental to this therapy is a person's capacity to set goals for rehabilitation. To date, no studies have assessed goal setting in early-stage Parkinson's disease dementia or dementia with Lewy bodies. Semistructured interviews were carried out with 29 participants from an ongoing trial of cognitive rehabilitation for people with these conditions. Here, we examined the goal statements provided by these participants using qualitative content analysis, exploring the types and nature of the goals set. Participants' goals reflected their motivations to learn new skills or improve performance in areas such as technology-use, self-management and orientation, medication management, and social and leisure activities. These results suggest that goal setting is achievable for these participants, provide insight into the everyday cognitive difficulties that they experience, and highlight possible domains as targets for intervention. The trial is registered with ISRCTN16584442 (DOI 10.1186/ISRCTN16584442 13/04/2015).

## 1. Introduction

At least 80% of people diagnosed with Parkinson's disease (PD) for more than 20 years develop dementia [1, 2]. The clinicopathological profile of this PD dementia (PDD) is similar to that of dementia with Lewy bodies (DLB [3, 4]), a condition whereby cognitive dysfunction antedates parkinsonism symptoms. These two syndromes share an almost indistinguishable neuropsychological profile, characterised by attentional and executive deficits alongside visuospatial and memory impairments [5, 6]. Other neuropsychiatric symptoms, such as hallucinations, depression, anxiety and apathy, may also emerge in these conditions [7]. Awareness of cognitive problems has received little research attention in both disorders. Nonetheless, some studies have indicated that people with PD may overestimate their perceived performance on cognitive tasks and for measures of daily living activities, when compared with informant ratings [8–11]. The negative impacts of neuropsychiatric symptoms on the wellbeing of people with these dementias and their caregivers, as well as on the broader community, have been noted [12–18]. Health services are required to support the functional independence of people with PDD and DLB through monitoring and managing their cognitive and behavioural changes in the context of increasing physical deterioration. Pharmacological treatments for cognitive symptoms are available and include cholinesterase inhibitors (e.g., rivastigmine) and glutamate receptor antagonists (e.g., memantine). These drugs, however, have been associated with adverse side-effects, such as increased tremor [19]. Furthermore, their use does not always correspond to improvements in functional independence or caregiver burden [20].

Nonpharmacological strategies may offer an alternative approach to the management of neuropsychiatric symptoms in these conditions. Recent efforts to assess the efficacy and feasibility of physical and psychological interventions (e.g., cognitive training, physical exercise, and participation in leisure or social activities) on neuropsychological and disease outcomes in people with PD without dementia have shown positive, albeit, limited results (for review, see [21, 22]). To date, no intervention studies have included people with PDD or DLB and thus the generalisability of these findings to these patient populations is restricted. In response to this gap, a pilot trial, entitled “Cognitive Rehabilitation for Parkinson's disease dementia: a pilot randomised controlled trial (CORD-PD),” assessing the application and potential efficacy of cognitive rehabilitation (CR) for people with mild to moderate PDD and DLB, is currently underway [23]. This randomised controlled trial (RCT) will compare CR against an active control condition (relaxation therapy, RT) and treatment as usual (TAU), using participants' ratings of, and satisfaction with, performance in relation to goals identified through an interview conducted by the researcher with the person with PDD or DLB and, if available, a primary caregiver. The objective of CR is to promote independence in the person with early-stage dementia by developing and supporting the use of strategies to circumvent the effects of cognitive difficulties. It uses an individualised approach whereby personally relevant goals are devised and implemented according to the individual's needs and abilities. The intervention is delivered by a trained therapist who adopts evidence-based methods and strategies appropriate to the nature of the cognitive goal set. These methods might include compensatory strategies (such as calendars, diaries, or reminders) and/or restorative approaches (such as mnemonics and spaced retrieval) to retain learned information and improve memory recall. The efficacy of CR, compared with RT and TAU, for people with early-stage Alzheimer's disease (AD) has been demonstrated in a single-centre RCT [24]. A large multicentre trial assessing the clinical- and cost-effectiveness of CR as an intervention for people with AD, vascular, or mixed dementia is underway. This research is not recruiting people with PDD or DLB due, in part, to their distinct motor symptoms [25]. The CORD-PD pilot trial will aim to assess the application of CR in the context of physical disability in these conditions.

Fundamental to the CR procedure is successful goal setting. This process involves an individual's capacity to identify areas of difficulty and devise goals for improvement, as well as rate and monitor performance and/or satisfaction with performance in relation to each selected goal. It is not yet known whether people with PDD and DLB can engage in effective goal setting for these purposes. Here, we explore the ability of people with mild to moderate PDD and DLB to set goals for CR. We will examine what types of goals people with PDD and DLB set and deem as important, as well as the nature and themes of their goal statements. A comparison of goal performance ratings from the perspectives of PDD and DLB participants and their caregivers will also be conducted.

## 2. Methods

### 2.1. Participants

Participants were recruited from Movement Disorder Clinics and Memory Services within the Betsi Cadwaladr University Health Board (BCUHB), North Wales, UK. Caregivers were invited to participate with the PDD or DLB participant's permission. Patient participants had a diagnosis of PD, PDD, or DLB according to consensus criteria, an Addenbrooke's Cognitive Evaluation-III (ACE-III, [26]) score of ≤82, and presented with early- to midstage dementia according to their clinician. Exclusion criteria included a diagnosis of any other significant neurological condition; major psychiatric disorder, including depression, which is not related to their Parkinson's disease; and unstable medication use for their physical or cognitive symptoms.

### 2.2. Design

Participants were recruited to an ongoing single-blind pilot trial which aims to assess the feasibility and efficacy of CR for people with PDD and DLB. This trial, CORD-PD, compares CR with TAU and an active control condition, RT. For further information regarding CORD-PD, please see the study protocol [23]. At the baseline visits, participants identified up to three rehabilitation goals and rated their performance and satisfaction with these goals, using the Bangor Goal Setting Interview (BGSI, [27]). Here, we explore the nature and types of goals identified by participants recruited so far to the study, in order to indicate the extent to which people with PDD and DLB are able to generate goals as well as what kind of goals they perceive as personally relevant and meaningful. We compare PDD and DLB participants' ratings to their caregivers' ratings for perceived goal performance from the baseline visits (i.e., prior to randomisation). Ethical approval for the study was obtained from the Wales Research Ethics Committee 5 (13/WA/0340). Consent procedures were conducted in accordance with the Declaration of Helsinki [28]; informed written consent was obtained from all participants prior to entry into the trial.

### 2.3. Measures

Only measures pertinent to the current analyses will be described. For a list and description of measures used in CORD-PD, please refer to the study protocol [23]. The BGSI [27] was used to identify and rate performance and satisfaction with goals set prior to randomisation (see Section 2.4). Severity of Parkinsonian symptoms was measured using the modified Hoehn and Yahr staging criteria [29]. Items from the Unified Parkinson's Disease Rating Scale-part three (UPDRS, [30]) were used to assess physical functioning, with higher scores indicating greater physical impairment (a maximum score of 92 was created from these item scores). The Hospital Anxiety and Depression Scale (HADS, [31]) assessed levels of anxiety and depression in the PDD and DLB participants and their caregivers (where available). Scores ≥ 11 on either subscale qualify for clinical levels of anxiety and depression symptoms [32]. Demographic information for participants with PDD or DLB and their caregivers was recorded at the baseline visits and includes age, sex, number of years of formal education, and marital status. IBM SPSS (version 22.0) was used to summarise these data [33].

### 2.4. Procedures

The BGSI was administered to all participants at the initial interviews prior to randomisation to the treatment groups. Specific goals were identified and selected through collaboration between the researcher, the person with PDD or DLB, and their caregiver (where available) over the course of these visits. At this point in time, prior to randomisation, the participants did not know if they would be allocated to the CR condition or the control conditions. The researcher remains blinded to participants' treatment allocations throughout their participation in the trial. At these visits, the researcher discusses the purpose of the CR intervention and asks participants to consider how they believe their cognitive difficulties affect their performance of everyday tasks, their enjoyment of activities and pastimes, and/or their participation in social activities. From these discussions, difficulties that the participant deems the most important and feels the most motivated to overcome are selected as the basis for possible goals. A minimum of two and a maximum of three goals are developed. Goals are further refined with help from the researcher and caregiver (if available) to produce a goal statement. Goal statements are specified according to SMART principles; they are specific, measurable, achievable, realistic, and timed [34]. For this study, if the participant stated a reason (i.e., a motivation) within the goal statement for why the goal was selected (e.g., “so my wife does not have to remind me”), this was included. Once the goal statement is agreed, the individual with PDD or DLB rates their current performance and satisfaction with their performance for these goals on a scale of 1–10, with 1 being unable to carry out or perform task/extremely dissatisfied with performance and 10 being able to carry out or perform task without difficulty/extremely satisfied with performance. The caregiver, where available, also rates the PDD or DLB participant's current goal performance using the same scale.

### 2.5. Qualitative Analysis

The goals generated by participants constituted the data set, to which a qualitative content analysis was applied [35]. Following the researcher's familiarisation with the data set through reading and rereading the data items (goals generated by participants), the data were coded for semantic content which organised the data items into two overarching categories: a “content” category, covering the types of goals or areas in which goals were set, and a “motivation” category, covering the motivations expressed by participants within the goal statement as a reason for selecting the goal. The data set was independently reviewed by a second researcher not associated with the current study but working on a separate CR trial [36]. The researchers consulted on the definitions of the final categories and subcategories until consensus was reached. A subsample of the data set (*n* = 23, 30.3%) was coded by the second researcher using the agreed framework. We used the Multiple Value Nominal Alpha (version 1.0) software programme [37] to compute Krippendorff's alpha coefficient of reliability [38]. The results of the interrater analysis showed excellent reliability (Krippendorff's alpha is 0.95), with percentage agreement being 95.7%. Where interrater discrepancies were found, these were discussed by the researchers and a consensus was reached.

## 3. Results

### 3.1. Participant Characteristics

Twenty-nine participants with PDD and DLB were recruited (25 PDD, 4 DLB). Median disease duration (time in years since diagnosis) for these participants was 4 years (1–21 years). The mean age for participants was 75.9 (SD = 6.7). The mean UPDRS score for participants was 30.3 (SD = 9.1). Participants were classified into the following Hoehn and Yahr stages: Stage 1 (*n* = 4, 13.8%); Stage 1.5 (*n* = 2, 6.9%); Stage 2 (*n* = 6, 20.7%); Stage 2.5 (*n* = 3, 10.3%); Stage 3 (*n* = 11, 37.9%); Stage 4 (*n* = 3, 10.3%). Six (21%) participants were female, in keeping with reports suggesting higher incidence rates of parkinsonism and PD in men [39]. The mean number of years of formal education for participants was 11.1 (SD = 1.7). Participants had a mean ACE-III score of 71.4 (SD = 7.6). Participants' mean performance on individual ACE-III domain scores are shown in Table 1. Participants had mean HADS mood scores for symptoms of anxiety and depression of 7.6 (SD = 4.1) and 7.0 (SD = 3.7), respectively. Six (20.7%) and three (10.3%) participants qualified for clinical levels of anxiety and depression, respectively, according to the criteria.

Twenty-six caregivers were recruited. The mean age for caregiver participants was 69.6 (SD = 10.6). Twenty-one (80.8%) of the caregiver participants were female. The majority of caregivers were spouses or partners of the person with PDD or DLB (*n* = 22, 84.6%), while the remaining caregivers were adult children of the person with PDD or DLB. Caregivers' mean HADS mood scores for symptoms of anxiety and depression were 4.8 (SD = 2.8) and 3.8 (SD = 2.4), respectively. Only one caregiver showed clinical levels of anxiety according to the criteria.

### 3.2. Perceived Goal Performance and Satisfaction with Goal Performance

Table 2 shows PDD and DLB participants' and caregivers' mean ratings for goal performance on the separate goals. Two caregivers refused to rate their partner's performance on goal 1 and goal 2, respectively, because they were not aware until the interview that their partner experienced difficulties with these tasks (these goals related to reading and word-finding difficulties). Table 2 also shows PDD and DLB participants' mean ratings for satisfaction with performance. Mean ratings were in the low range of the 1–10 performance scale which are appropriate for baseline ratings before the introduction of an intervention and indicative of good interviewing practice by the researcher. A paired sample *t*-test revealed that PDD and DLB participants' mean ratings for perceived goal performance (M = 3.1, SD = 1.6) were significantly higher than the caregivers' mean ratings for PDD and DLB participants' performance (M = 2.4, SD = 1.5) [*t*(65) = 4.6, *p* < 0.001, *d* = 0.5].

### 3.3. Qualitative Analysis

The 29 PDD and DLB participants identified 76 goals in total, with 18 participants generating three goals each and 11 participants generating two goals each.  Table 3 shows the final categories and subcategories identified from the data set and the frequency with which these subcategories were endorsed by participants. The next sections delineate these categories and subcategories with examples of participants' goal statements.

#### 3.3.1. Content Category

*(1) Technology.* Most of the goal statements surrounded themes of technology-use, such as learning or relearning how to use personal computers, tablets, or mobile phones:

 “*I will be able to use email.*”
 “*Use the word processor on my computer to write short documents.*”
 “*I will be able to use a mobile phone to send and receive text messages.*”

*(2) Leisure Pursuit and Maintenance.* Another common theme was maintaining existing or starting new activities or pastimes:

 “*I will be able to read for half an hour in a day and remember what I read.*”
 “*I will be able to prepare a simple pasta dish.*”
 “*I will create a portfolio of twenty-five photos.*”

*(3) Medication Management.* Several participants spoke of difficulties with managing their medication on their own and were motivated to devise or refine their existing medication schedules in accordance with prescribed times and dosages:

 “*Devise a medication schedule to remember to take my medication on time.*”
 “*Refine medication system so that I will be able to take medication on time at the right dosage when at home and during the day.*”

*(4) Self-Management and Orientation.* Some participants indicated an eagerness to manage their own time and daily activities or to orientate themselves in time or place:

 “*Within two months, I would like to devise a personalised system to keep track of diary appointments and activities of the care home.*”
 “*I will learn to navigate different rooms of my house without getting lost.*”
 “*Know the date and keep track of time (morning, mid-day, evening) during the day.*”

*(5) Important Items.* Some participants expressed frustration at misplacing everyday items and wanted to use strategies to retrieve these:

 “*I will remember where I placed my personal items, such as my wallet and glasses.*”

*(6) Social Interaction and Communication.* A few participants expressed social embarrassment at forgetting people's names and losing their train of thought or “words” when in conversation with people. Three participants noted their word retrieval issues and wanted to understand and overcome these:

 “*Identify the reason for my word-finding difficulties and learn strategies to circumvent these failures.*”
 “*I will remember approximately half of the names of my team-members in my bowls club.*”
 “*I will learn strategies to assist with retrieving the correct word in conversations with people.*”

*(7) Anxiety Management.* Two participants were strongly concerned about the anxiety attacks they were experiencing and wanted to learn strategies to help them cope with these episodes. One participant experienced separation anxiety when away from her husband and wanted to remind herself to use distraction tactics during these separations:

 “*I will be able to develop and use strategies to overcome or cope with my anxiety attacks.*”

#### 3.3.2. Motivation Category

*(1) Relationships.* Participants often expressed a desire to use technology to maintain family and social relationships:

 “*I would like to learn how to use my mobile phone so that I can call family members for either social reasons or emergencies.*”
 “*I will use Skype on the computer to be able call a friend or family member.*”

One participant indicated he was motivated to dedicate more time to his reading in order to benefit his social interactions, since his wife complained that he no longer contributes to conversations:

 “*I will dedicate one hour per day to read about my topic of interest (space travel) so that I can have conversations with people about it.*”

*(2) Knowledge Pursuit.* Several participants wished to acquire skills in novel technology in order to pursue knowledge or special interests:

 “*I will be able to use the laptop to access the internet to read news articles.*”
 “*I will learn how to use the internet to research bowling techniques.*”
 “*I want to be able to find information (general knowledge, current affairs) online using my desktop and iPad, within two months.*”

*(3) Family Benefit.* Some goal statements indicated a desire to benefit family members or relieve them of care duties:

 “*I will remember to record the events of the day (such as visits or appointments) to keep track of daily events so that my daughter is informed.*”
 “*I will select and retrieve items of clothing to wear for the next day rather than rely on my wife.*”
 “*I will refine my medication system so that I can take my pills on time without being prompted by my wife.*”

## 4. Discussion

The findings presented here indicate that goal setting for CR is achievable for people with PDD and DLB. They also highlight participants' motivation to acquire skills in technology, start or maintain pastimes, and manage their own time and daily activities despite their cognitive difficulties. Difficulty in complying with medication regimes was another main category that emerged from the analysis and poses particular clinical implications for these patient groups. On average, people with PDD and DLB rated their current performance for personalised goals in the low range, indicating their awareness of difficulties with these tasks. Nonetheless, relative to informant ratings, people with PDD or DLB rated their performance as significantly better for most of the goals prior to the start of the intervention phase.

To our knowledge, this is the first study to assess goal setting for CR in PDD and DLB. Earlier research has examined goal setting in 26 people with PD without dementia [40]. This study classified participants' goals under domains of executive function in preparation for an intervention which comprised strategic executive training. The study reported that the top three domains in which PD participants set goals were for “regulation” (goals relating to monitoring and the execution of tasks), “planning” (goals relating to processes preceding the execution of a task), and “initiative” (goals that apply to motivating oneself to start activities or encourage a positive change in activity level). Moreover, relative to participants with acquired brain injury, PD participants set significantly more goals associated with “time management,” which they defined as goals relating to deficits in estimating time. Our findings somewhat align with the results of this work. In particular, participants in our sample showed an eagerness to orientate themselves in time as well as plan and organise their leisure and clinical appointments themselves, through the use of diaries and calendars. Designing or improving their medication scheduling, as well as increasing their independence with this task, would require them to adopt strategies (e.g., devising a checklist and using an alarm system) to assist with planning and self-monitoring. The selection of such goals by our participants may underlie impairments in these processes leading to cognitive difficulties, particularly for the prospective aspects of memory. Suboptimal medication adherence in people with PD has been highlighted by systematic research [41] which suggested that several factors, such as mood disorder, cognition, and scheduling complexity (e.g., polypharmacy), contributed to lack of compliance. Our participants identified their cognitive difficulties as the main source of their nonadherence. This finding has important implications for practitioners assisting people with PD experiencing cognitive difficulties and suggests that they may require more routine monitoring by clinical staff.

Many participants indicated a desire to maintain or improve their leisure activity levels, possibly in response to a self-acknowledged apathy in relation to their usual interests or a deterioration in their abilities. The desire to learn new skills, particularly in the area of novel technologies, was most commonly reported. These skills were often selected for the purpose of wanting to research current affairs and knowledge or remain in contact with family members through social media. A challenge in selecting these goals during the interview phase was that some activities required a physical output (such as bowls, cooking, or typing while using a computer) and* both* cognitive and physical impairments contributed to reduced functioning in these areas. It was thus important to manage participants' expectations regarding possible outcomes of the proposed therapy and emphasise support for their cognitive difficulties with these tasks. People with early PDD and DLB showed an awareness of the impact of their cognitive difficulties on their social outcomes and relationships, with some participants electing to focus on strategies to help them remember peoples' names or circumvent word retrieval difficulties when in conversation. Further, some participants showed a desire to improve performance on certain tasks in order to diffuse burden or responsibility of care for their relatives.

Interestingly, participants with PDD and DLB rated their goal performance in the low range, but as significantly better relative to informant-reports, suggesting that while they show awareness of the presence of their cognitive difficulties they might underestimate the severity of these impairments or their impact on daily life. Impaired awareness in people with PD for cognitive performance has been suggested previously [8, 10, 11], but this remains a neglected area of research within the condition. It is possible that these findings might be explained by caregivers' underestimation of their partners' abilities due to psychological responses or biases. As a group, the caregivers in our sample did not show clinical levels of anxiety or depression, but other factors, such as their partner's cognition, neuropsychiatric symptoms and/or disease severity, perceived burden, time commitment or relationship quality with the person with PDD or DLB, might have influenced their appraisals. Such factors have been suggested to influence informant-reports of performance for people with AD [42–46].

This study is not without its limitations. Our sample size is small which might compromise the representativeness of our participant groups and the generalisability of these findings to the wider PDD, DLB, and respective caregiver populations. Similarly, the PDD and DLB participants were not severely cognitively or physically disabled which might have influenced the nature or types of goals selected. Given the restricted sample size, it is difficult to determine whether specific impairments in cognitive functions (e.g., attention or memory as measured by the ACE-III) influenced goal setting or selection. Moreover, other factors such as predominant physical symptoms (e.g., tremor, postural imbalance, and bradykinesia), levels of apathy, and/or personality variables, which were not assessed here, might have also influenced this process. Understanding the impact of such factors on goal setting in these patient groups, and others, should be explored in larger future studies. The goal setting interview constituted a semistructured approach with discussions directed around participants' everyday functioning, social interaction and leisure activities; this might have excluded other areas important to participants. Some participants proffered their motivations for selecting particular goals within the goal statement, while others did not. Therefore, some motivations behind participants' goal setting might have been omitted or implied but not coded within the current analysis. Participation of the caregiver in the goal setting session, while benefitting these discussions, may have also influenced participants' expressed priorities for rehabilitation.

## 5. Conclusions

Despite several limitations, this study demonstrates that people with early-stage PDD and DLB can generate suitable goals for rehabilitation. It describes the everyday cognitive difficulties that these individuals experience and highlights areas of difficulty for targeted intervention. The next steps in the ongoing trial will be to explore whether implementation of the CR intervention is feasible for these patient groups and whether it shows efficacy in producing improvements for ratings of performance and satisfaction with cognitive goals.

## Figures and Tables

**Table 1:** Mean performance on ACE-III domain scores.

ACE-III domain (max score)	Patients with PDD and DLB			
	M	SD	Min–max	*N*
Total (100)	71.4	7.6	48–81	29
Attention (18)	14.5	2.4	7–18	29
Memory (26)	14.7	3.4	9–21	29
Fluency (14)	7.7	2.9	0–11	29
Language (26)	22.6	2.1	18–26	29
Visuospatial (16)	11.8	1.2	8–15	29

**Table 2:** Mean ratings for PDD and DLB participants' goal performance and satisfaction.

Goal	PDD and DLB performance ratings				PDD and DLB satisfaction ratings				Caregiver ratings for PDD and DLB participants' performance			
	M	SD	Min–max	*N*	M	SD	Min–max	*N*	M	SD	Min–max	*N*
1	3.1	1.9	1–6	29	3.3	1.9	1–7	29	2.2	1.3	1–5	24
2	3.2	1.6	1–5	29	3.3	1.7	1–7	29	2.6	1.4	1–5	24
3	2.8	1.5	1–5	18	3.3	2.0	1–8	17	2.3	1.9	1–7	15

**Table 3:** Categories of goal statements.

“Content” category	Definition	Count
Technology	Learn or relearn how to use a technological device or software	34
Activities or pastimes maintenance	Start or maintain an activity or pastime	19
Medication management	Develop a medication schedule or remember to take medication on time or at correct dosage	10
Self-management and orientation	Orientate self in time or place; plan his/her schedule; record information/events of the day; organise daily functional activities or environment	10
Important items	Remember where he/she placed important items or remember to take important items with him/her to events	2
Social interaction and communication	Remember names of people and be able to maintain a conversation (e.g., address word finding and concentration difficulties)	6
Anxiety management	Learn strategies to manage anxiety or panic attacks	2

“Motivation” category	Definition	Count

Relationships	Motivated to maintain family and social relationships	7
Knowledge pursuit	Motivated to acquire knowledge about current affairs or research personal topics of interest	6
Family benefit	Motivated to reduce burden for family or because it is important to family	7
